# Artificial dispersion via high-order homogenization: magnetoelectric coupling and magnetism from dielectric layers

**DOI:** 10.1098/rspa.2013.0240

**Published:** 2013-10-08

**Authors:** Yan Liu, Sébastien Guenneau, Boris Gralak

**Affiliations:** CNRS, Ecole Centrale Marseille, Aix-Marseille Université, Institut Fresnel, UMR 7249, 13013 Marseille, France

**Keywords:** homogenization, transfer matrix, Sophus Lie theorem, magnetoelectric coupling

## Abstract

We investigate a high-order homogenization (HOH) algorithm for periodic multi-layered stacks. The mathematical tool of choice is a transfer matrix method. Expressions for effective permeability, permittivity and magnetoelectric coupling are explored by frequency power expansions. On the physical side, this HOH uncovers a magnetoelectric coupling effect (odd-order approximation) and artificial magnetism (even-order approximation) in moderate contrast photonic crystals. Comparing the effective parameters' expressions of a stack with three layers against that of a stack with two layers, we note that the magnetoelectric coupling effect vanishes while the artificial magnetism can still be achieved in a centre-symmetric periodic structure. Furthermore, we numerically check the effective parameters through the dispersion law and transmission property of a stack with two dielectric layers against that of an effective bianisotropic medium: they are in good agreement throughout the low-frequency (acoustic) band until the first stop band, where the analyticity of the logarithm function of the transfer matrix (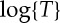) breaks down.

## 1. Introduction

There is a vast amount of literature on the homogenization of moderate contrast periodic structures with the classical effect of artificial anisotropy. A less well-known effect is artificial magnetism and low-frequency stop bands through averaging processes in high-contrast periodic structures [1–5].

In layman terms, O'Brien and Pendry observed in 2002 that rather than using the LC-resonance of conducting split ring resonators to achieve a negative permeability as an average quantity [1,6], one can design periodic structures displaying some Mie resonance, e.g. by considering a cubic array of dielectric spheres of high-refractive index [2]. These resonances give rise to the heavy-photon bands in photonic crystals that are responsible for a low-frequency stop band corresponding to a range of frequencies wherein the effective permeability takes extreme values [4]. The so-called high-contrast homogenization [5] predicts this effect, which is associated with the lack of a lower bound for a frequency-dependent effective parameter deduced from a spectral problem reminiscent of Helmholtz resonators in mechanics.

In this paper, we achieve such a magnetic activity without resorting to a high-contrast material. The route we propose is based upon a homogenization approach for high frequencies, i.e. when the period of a multi-layered structure approaches the wavelength of the applied field. The extension of classical homogenization theory [7–9] to high frequencies is of pressing importance for physicists working in the emerging field of photonic crystals and metamaterials [10], but applied mathematicians also show a keen interest in this topic [3,11,12], where the periodic structures at sub-wavelength scales (*λ*/10 to *λ*/6) [2,6] can clearly be regarded as almost homogeneous.

The tool of choice for our one-dimensional model is the transfer matrix method, which allows for analytical formulae as shown in §2, a high-order homogenization (HOH) method is proposed wherein the Baker–Campbell–Hausdorff formula (the BCH formula, an extension of Sophus Lie theorem) was implemented; we stress that ideas contained therein can be extended to two-dimensional periodic structures like lamellar gratings, see [13], because our model extends those of Rytov [14]. Importantly, we not only achieve magnetic activity in moderate contrast dielectric structures, but also unveil some artificial magnetoelectric coupling (a particular case of bianisotropy) in §3, where a multi-layered stack with an alternation of two dielectric layers is considered. As proposed by Pendry, the bianisotropy is yet another route towards negative refraction [15]. However, it is noted that the artificial magnetoelectric coupling vanishes in a periodic stack with centre symmetry, where an extension of the HOH method applied to a stack with *m* (≥3) alternative layers is explored in §4. Furthermore, a correction factor is investigated in §5 to estimate the asymptotic error, which is proportional to 1/*n*^*p*^ with *p* the approximation order. We then numerically check the effective parameters through the dispersion law and transmission property between the multi-layers and the effective medium in §6: a good agreement throughout the low frequency band up to the first stop band illustrates the equivalence between the structured dielectric medium and its effective bianisotropic counterpart. We finally consider a frequency power expansion of the transfer matrix, which is analytical in the whole complex plane in §7 and draw some concluding remarks in §8.

## 2. Mathematical set-up of the problem

**Figure 1.**
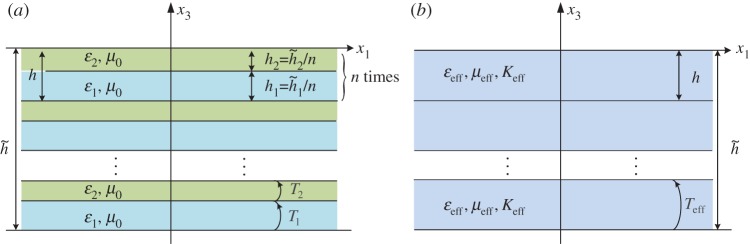
(*a*) Schematic of a multi-layered stack consisting of an alternation of two homogeneous dielectric layers of permittivities *ε*_1_, *ε*_2_ and thicknesses 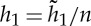, 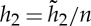, with *n* the number of unit cells. The thickness of the stack 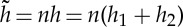 is comparable with the wavelength *λ* of the applied field 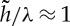. When *n* is large enough, 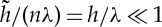, i.e. *ωh*/(2*πc*_0_)≪1 with *c*_0_ the velocity of light in vacuum. (*b*) Effective medium described by anisotropic tensors of permittivity, permeability and magnetoelectric coupling, with thickness 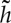. (Online version in colour.)

Figure 1*a* shows a schematic of periodic multi-layered stack with a unit cell made of two homogeneous dielectric layers 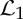 and 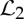 of respective thicknesses *h*_1_ and *h*_2_ (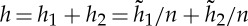), where *n* stands for the number of the unit cells in the stack. The permittivities and the permeabilities of the two layers are denoted by *ε*_1_, *ε*_2_ and *μ*_1_=*μ*_2_=*μ*_0_ (with *μ*_0_ the permeability of vacuum), respectively. It should be noted that the whole thickness of the stack 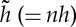 is a constant and it is comparable with the wavelength *λ* of the applied field as 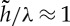. Starting from the case when *n*=1, the stack is the most simple structure consisting of only two dielectric layers, in which case the effective medium theory [16–18] cannot be applied when *h*/*λ*≈1. However, if we increase *n* to a large enough constant, then the system contains *n* times smaller unit cells, with a thickness much smaller than the wavelength of the applied field (e.g. *h*/*λ*≪1). Hence, a homogeneous medium with permittivity *ε*_eff_, permeability *μ*_eff_ and magnetoelectric coupling *K*_eff_ shown in figure 1*b* can be assumed to behave as an effective medium for such a multi-layer. The approximation of the multi-layer by the effective medium will be all the more accurate than *n* is large, a fact which will be proved in the following sections.

### (a) Time-harmonic Maxwell's equations

At the oscillating frequency *ω*, the electric and magnetic fields **E** and **H** are related to the electric and magnetic inductions **D** and **B** through the time-harmonic Maxwell's equations
2.1
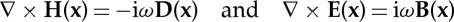
and the constitutive relations for non-magnetic isotropic dielectric media,
2.2
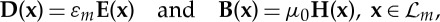
with *ε*_*m*_ the permittivity in the *m*th homogeneous layer located in the domain 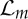 of 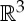.

Then, a Fourier decomposition is introduced for both electric and magnetic fields as
2.3
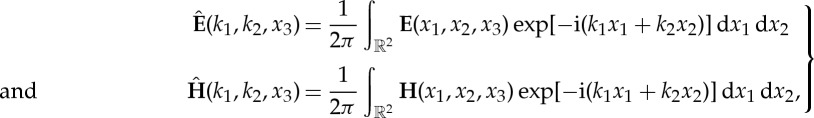
with *k*_1_ and *k*_2_ the projections of wavevector **k** on *x*_1_ and *x*_2_ axes, respectively; the wavevector for an oblique plane wave is denoted by **k**=(*k*_1_,*k*_2_,*k*_3_).

Applying the decomposition (2.3) to (2.1) and (2.2), we derive an ordinary differential equation (involving a 4×4-matrix and a four-component column vector) [19]
2.4
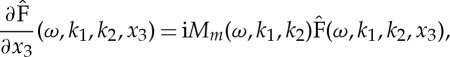
where 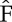 is a column vector containing the tangential components of the Fourier-transformed electric field 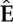 and magnetic field 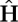, i.e. their components along *x*_1_ and *x*_2_ axes.

Here, in order to simplify subsequent calculations, we define a new set of coordinates (*x*_∥_, *x*_⊥_, *x*_3_): The component *x*_∥_ is along the direction of wave vector ***k***=(*k*_1_,*k*_2_), *x*_⊥_ is along ***k***′=(−*k*_2_,*k*_1_), which is perpendicular to *x*_∥_. In other words, the new set of coordinates is a rotation of the previous coordinates around the *x*_3_-axis. The change of coordinates from (*x*_1_, *x*_2_, *x*_3_) to (*x*_∥_, *x*_⊥_, *x*_3_) can be expressed as
2.5
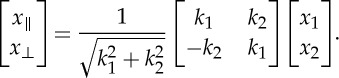
Note that, thanks to the symmetry of the geometry, the parameters of the multi-layer are invariant under this transformation [20].

Hence, (2.4) can be recast in the new coordinate system as
2.6
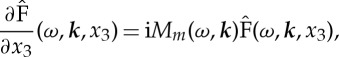
with the column vectors
2.7
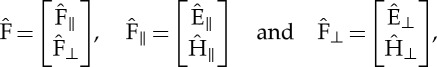
where 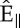, 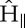 and 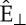, 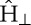 are the components of the electric and magnetic fields along the *x*_∥_-axis and the *x*_⊥_-axis, respectively.

Correspondingly, *M*_*m*_ is a 4×4 matrix, the components of which can be expressed as
2.8
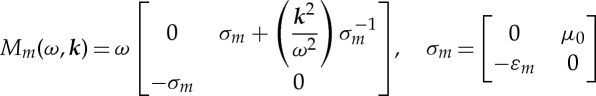
where ***k***^2^=***k***⋅***k*** and ***k*** is the two-dimensional component of the three-dimensional wavevector **k** after projection in the plane (*x*_1_,*x*_2_).

The matrix *M*_*m*_(*ω*,***k***) is independent of *x*_3_ in each homogeneous layer, the solution of (2.6) in the layer 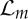 is simply
2.9
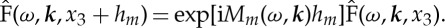
The exponential above is well defined as a power series of matrix *M*_*m*_(*ω*,***k***) and defines the transfer matrix in the *m*th layer of thickness *h*_*m*_, which relates the total tangential components of electric and magnetic fields at the two ends of the slab. Note that this definition only differs by a change of basis from an equivalent definition of the transfer matrix which relates the upward and the downward propagation fields at the two ends of the slab. As this power series has an infinite radius of convergence, the transfer matrix
2.10
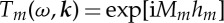
is analytical with respect to the three independent variables *ω*, *k*_1_ and *k*_2_. For an arbitrary permittivity profile (with the classical assumption of upper and lower bounds of permittivity greater than *ε*_0_ (the permittivity of vacuum) uniformly in position **x** and the number of layers *n*), analyticity is proved using a Dyson expansion [21].

### (b) Main homogenization result

From the knowledge of the transfer matrix in multi-layered stacks, we deduce that the most general expression of the transfer matrix [22] can be obtained from the following homogenized constitutive equations
2.11
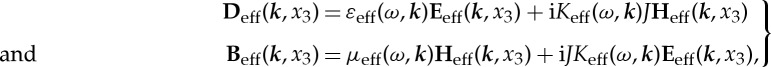
where *ε*_eff_ and *μ*_eff_ are tensors of rank 2, which represent, respectively, the (anisotropic) effective permittivity and permeability as follows:
2.12
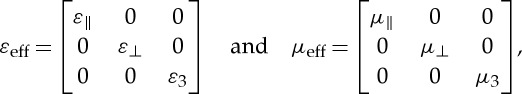
of the effective medium, the matrix *J* corresponds to 90^°^ rotation around the *x*_3_-axis, and *K*_eff_ is the bianisotropic parameter measuring the magnetoelectric coupling effect
2.13
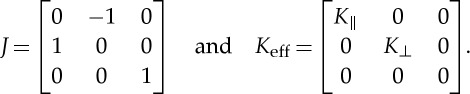
Now, applying (2.3) to (2.1) and (2.11), we obtain
2.14
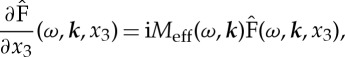
with matrix
2.15
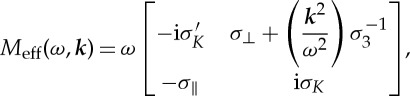
where the 2×2 blocks are defined by
2.16
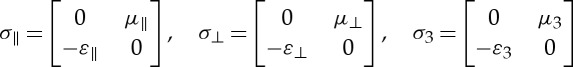
and
2.17
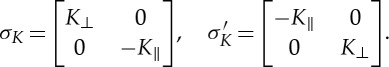
These parameters are all unknowns at this stage which we derive from a homogenization algorithm. The transfer matrix is correspondingly,
2.18
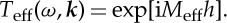


## 3. High-order homogenization algorithm for multi-layered stack

As we have derived the transfer matrix for the multi-layered stack and postulated its structure for the effective medium in the previous section, it follows that the description of the homogenization procedure shown in figure 1 can be expressed as
3.1
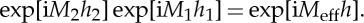
This means that the two structures should exhibit the same transmission property, where *M*_eff_ is the unknown to be calculated. Note that the left-hand side of (3.1) is a product of two exponential functions, which can be approximated by introducing the BCH formula (an extension of the Sophus Lie theorem, see [23]). In mathematics, the BCH formula is concerned with
3.2
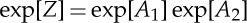
with *A*_1_ and *A*_2_ some square matrices. An analytical expression for *Z* is
3.3
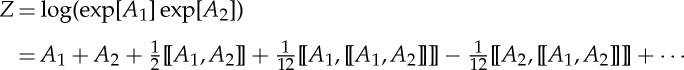
where [[*A*_1_,*A*_2_]]=*A*_1_*A*_2_−*A*_2_*A*_1_ is the commutator of *A*_1_ and *A*_2_, the product of which is non-commutative with [[*A*_2_,*A*_1_]]=−[[*A*_1_,*A*_2_]]. Here, we denote *A*_1_+*A*_2_ in (3.3) as the zeroth-order approximation *Z*^(0)^ for *Z*, which corresponds to the classical homogenization result; [[*A*_1_,*A*_2_]]/2 is the first-order correction *Z*^(1)^, [[*A*_1_,[[*A*_1_,*A*_2_]]]]/12−[[*A*_2_,[[*A*_1_,*A*_2_]]]]/12 the second-order correction *Z*^(2)^, and so on. The *m*th (*m*≥1) order approximation for *Z* is then defined as 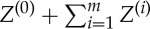.

From (3.3) and (3.1), we have
3.4
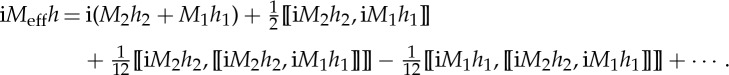
Furthermore, the expressions for the effective parameters in (2.12) and (2.13) can be derived by comparing the two matrices on the left- and right-hand sides of (3.4).

First, we consider the zeroth-order approximation in (3.4), it yields *M*_eff_≈*M*_1_*f*_1_+*M*_2_*f*_2_ with the filling fractions *f*_1_(=*h*_1_/*h*) and *f*_2_(=*h*_2_/*h*), respectively, and the effective parameters are
3.5
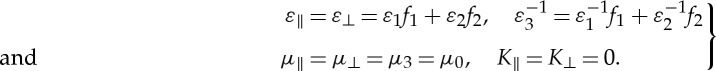
They are identical to the effective parameters obtained in [17,18,24–26] by classical homogenization: the effective permittivity and permeability are equal to their average within two dielectric layers, while the magnetoelectric coupling is zero.

If we go further by taking the first-order approximation, we obtain
3.6
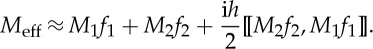
As both *M*_1_ and *M*_2_ are off-diagonal matrices, then their commutator leads to a diagonal matrix, the components of which correspond to those of *M*_eff_ in (2.15), i.e.
3.7
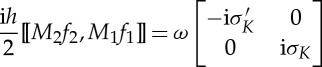
where
3.8
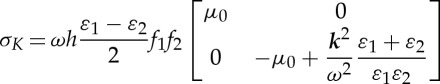
provides the first-order correction to the leading order approximation (classical homogenization) in (3.5). This first-order correction is encompassed in the following magnetoelectric coupling coefficients:
3.9
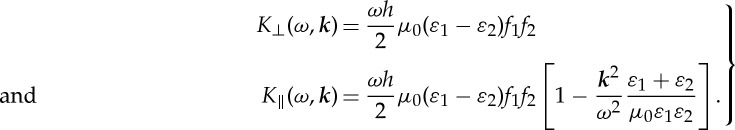
Note that the tensor *K*_eff_ is not only frequency-dependent but also exhibits spatial dispersion. The latter leads to *K*_∥_≠*K*_⊥_ when ***k***≠**0**.

Furthermore, if we consider the second-order correction, a term with ‘double commutator’ will appear in the asymptotic expansion
3.10
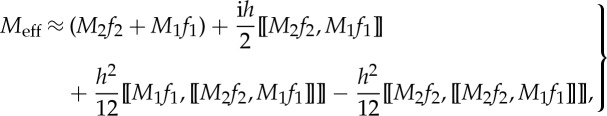
with commutator of *M*_1_ and *M*_2_ given in (3.7), and double commutator
3.11
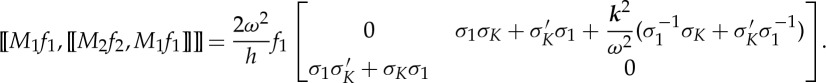
According to the definitions of *σ*_*m*_ in (2.8), *σ*_*K*_ and *σ*′_*K*_ in (2.17), we have
3.12
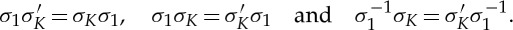
Thus, (3.11) reduces to
3.13
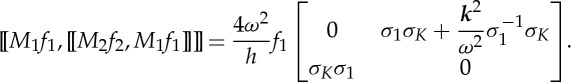
Similar equalities hold for [[*M*_2_*f*_2_,[[*M*_2_*f*_2_,*M*_1_*f*_1_]]]]. Hence, the terms arising from ‘double commutator’ lead to
3.14
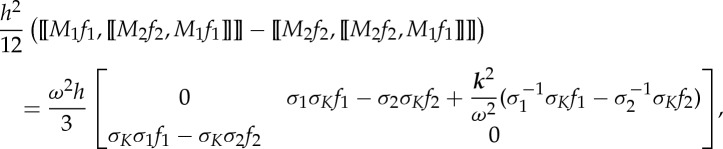
which is again an off-diagonal matrix. Substituting (3.14) into (3.10) and comparing with the form of matrix *M*_eff_ in (2.15), we find
3.15
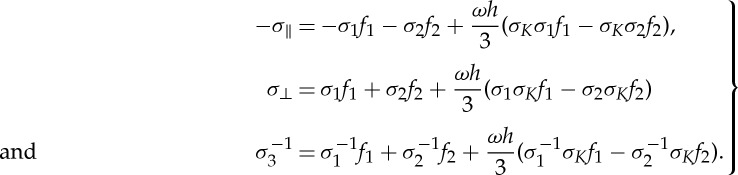
Finally, the expressions of *ε*_eff_, *μ*_eff_ are as follows:
3.16
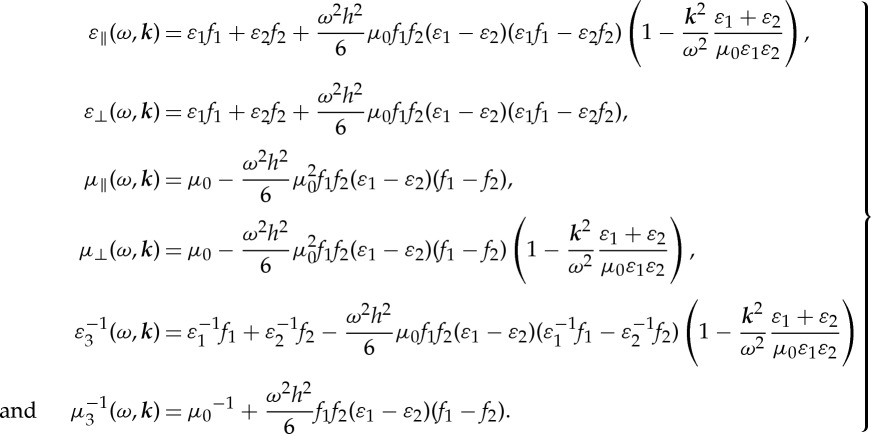
All the effective parameters are frequency-dependent and with spatial dispersion. Note that, the expressions above remain well-defined in the limit 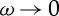 since the ratio ***k***/*ω* is fixed by the angle of incidence. These expressions turn out to be equivalent to the ones reported in [14,25,27], where the effective refractive index *n*_eff_ is expanded using a power series of period-to-wavelength ratio *Λ*/*λ*. Taking eqn (5) (s-polarized incidence is considered) in the paper of Gu & Yeh [27] as an illustration, the dispersion relation for a two-component layered medium is approximated by taking the fourth order of *O*[(*Λ*/*λ*)^2^] as
3.17
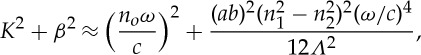
where *K* and *β* are the *z* and *x* components of the Bloch wavevector, *a* and *b* are the thicknesses of the alternating layers, *Λ*=*a*+*b* is the period, *n*_1_ and *n*_2_ are the indices of refraction of the corresponding layers, *c* the velocity of light in vacuum (*c*^−2^=*ε*_0_*μ*_0_), and
3.18
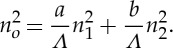
Comparing with the notation in our formulae, we have
3.19
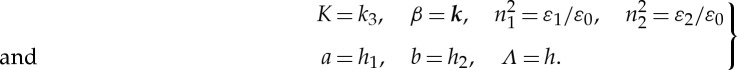
hence, *f*_1_=*a*/*Λ*, *f*_2_=*b*/*Λ*. Then (3.17) becomes
3.20
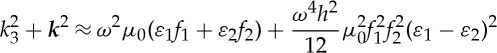
which contains the terms of order *ω*^2^ and *ω*^4^. On the other hand, for the effective medium in our HOH process, the dispersion relation of *k*_3_ versus *ω* is
3.21
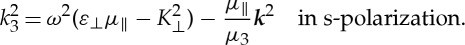
Let us substitute the expressions of effective parameters (3.16) into (3.21) and collect the terms up to order *ω*^4^ in the calculation process, we obtain the same formula as (3.20). Similar calculation applied to a p-polarized incident wave shows that again our homogenization provides exactly the same effective index. Hence, it is stressed that the effective parameters *ε*_eff_, *μ*_eff_ and *K*_eff_ identified using the HOH algorithm contain more information than the single effective index parameter in [14,25,27], e.g. artificial magnetism and magnetoelectric coupling from periodic dielectrics that is not captured in the derivation of the refraction index.

In the asymptotic process, we have noticed that magnetoelectric coupling comes from the odd-order approximation, while artificial magnetism and high-order corrections to permittivity emerge from the even-order approximation in (3.3). This can be explained in the following way: the matrix *M*_*m*_ (*m*=1,2) for the dielectric layer is off-diagonal, the terms of the odd-order correction usually contain odd commutators, hence a diagonal matrix will result, the components of which correspond to *K*_∥_ and *K*_⊥_. However, the terms of the even-order correction contain even commutators, hence the resulting matrix is always off-diagonal. Physically, this introduces artificial magnetism and high-order corrections to permittivity.

Moreover, these results are fully consistent with descriptions in terms of spatial dispersion [28,29] where, expanding the permittivity in power series of the wavevector, first order yields optical activity and second order magnetic response. The equivalence of these two descriptions (frequency and wavevector power series) is confirmed by considering a unit cell with a centre of symmetry, for example, a stack of three homogeneous layers (permittivity *ε*_*m*_ and thickness *h*_*m*_, *m*=1, 2, 3) with *ε*_3_=*ε*_1_ and *h*_3_=*h*_1_. Extending (3.3) to the case 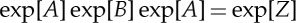 (see §4), it is found that *K*_eff_=**0**, and thus it is retrieved that both magnetoelectric coupling and optical activity vanish in a medium with a centre of symmetry [28].

The present expansion in power series of frequency provides a new explanation for artificial magnetism and magnetoelectric coupling. The analytical expressions (3.16) of effective parameters can be used to analyse artificial properties. In particular, we show from (3.16) that: artificial magnetism, previously proposed with high contrast [2,3,12], can be obtained with arbitrary low contrast; and magnetoelectric coupling, previously achieved in Ω-composites [30], can be present in simple one-dimensional multi-layers. Note that, one can obtain more accurate asymptotic expressions for the effective parameters with more terms in (3.9) and (3.16), by taking higher order approximations in (3.3).

Although this homogenized system has been studied by Ramakrishna & Lakhtakia [31], these authors assumed some magnetism and magnetoelectric coupling for the periodic multi-layered stack, whereas in our case these effects are deduced from a homogenization process (one might say *ex nihilo*). Moreover, these authors assumed that the matrix of magnetoelectric coupling was diagonal, which is not the case in this paper. It is, to the best of our knowledge, the first time these constitutive relations are derived, and we emphasize that the mathematical theorem invoked in this section (BCH formula) can be used to generalize our results to two-dimensional periodic structures [32], for example, as woodpiles [13]. In the sequel, we shall also investigate numerically the stop band properties of such a periodic stack of dielectrics and draw some illuminating parallels with the seminal paper by Pendry [15].

## 4. Extension of Baker–Campbell–Hausdorff formula for *m* layers

In §3, we have introduced our HOH algorithm for a periodic multi-layered stack consisting of an alternation of two dielectric layers, wherein the BCH formula is implemented. In this section, we investigate the extension of HOH to a stack with *m* layers in a unit cell, therefore a new form of BCH formula should be explored. We start with *m*=3, which means a multi-layered stack with an alternation of three dielectric layers is considered and the thickness of each layer in a unit cell is *h*_*m*_ with *m*=1,2,3. Then, the transfer matrix of one unit cell can be expressed as
4.1
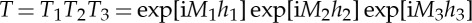
or in a more compact form
4.2
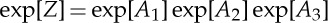
which defines a product of matrix exponentials. Obviously, it can be developed using an iteration of the BCH formula. First, we suppose
4.3
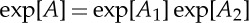
and *A* can be derived through (3.3)
4.4
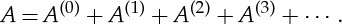
The term *A*^(0)^ is the zeroth-order approximation, while *A*^(*i*)^ represents the *ith* (*i*≥1) order correction and more precisely
4.5
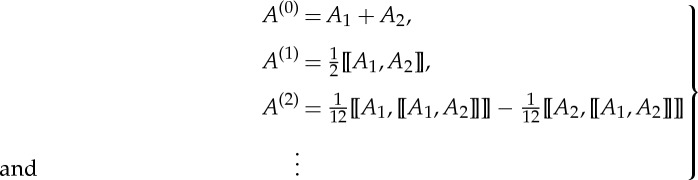
so that (4.2) takes the form:
4.6
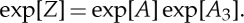
Using now the BCH formula for *Z*
4.7
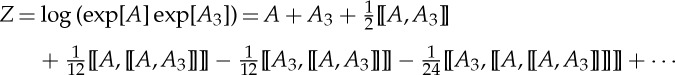
Here, we assume *Z*=*Z*^(0)^+*Z*^(1)^+*Z*^(2)^+⋯ , where *Z*^(*m*)^ is the *m*th (*m*≥1) order correction for *Z*. The zeroth-order approximation *Z*^(0)^ is simply the sum of *A*_1_, *A*_2_ and *A*_3_,
4.8
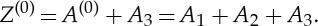
The first-order correction including a single commutator of these three matrices *A*_1_, *A*_2_ and *A*_3_ is
4.9
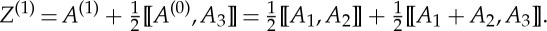
and the second order including a double commutator is
4.10
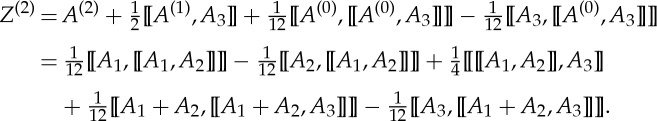
A similar algorithm holds for the third and higher orders, but it will not be further explored here.

As the BCH formula for (4.2) has been derived, one can easily perform the homogenization of a multi-layered stack with an alternation of three dielectric layers. Here, we assume that the third layer of the unit cell is identical to the first layer, i.e. *ε*_3_=*ε*_1_ and *h*_3_=*h*_1_, as well as *M*_3_=*M*_1_; taking (4.9) with *A*_1_=*A*_3_, we have *Z*^(1)^=**0**. It should be noted that all the odd-order corrections in the HOH asymptotics vanish, which can be attributed to the centre-symmetric property of the structure [22]. By contrast, even orders rule the approximation process in that case. Applying the formulae (4.8)–(4.10) to (4.1), one deduces the expressions for the effective parameters at the second-order approximation as follows:
4.11
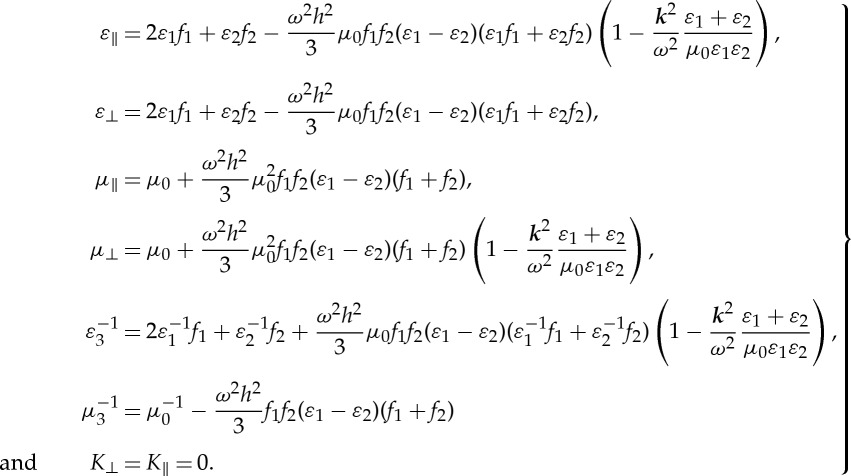
The effective magnetoelectric coupling tensor *K*_eff_ is equal to zero because *Z*^(2*p*+1)^=**0**, and only artificial magnetism and high order corrections to the permittivity persist. A similar calculation can be applied to higher order approximation, e.g. the expressions of these parameters at the fourth-order approximation under a normal incidence are discussed in [33].

So far, we have discussed the HOH asymptotic for a multi-layered stack consisting of an alternation of two layers as well as three layers; and the BCH formula has also been amended correspondingly. If we extend this asymptotic procedure to a more general case, i.e. we consider a stack with an alternation of *m* (≥3) layers, then the transfer matrix of a unit cell becomes
4.12
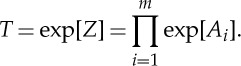
Once again, tedious iteration of BCH in (4.12) can produce all the formulae for different orders of approximation. Here, we just list the formulae from the zeroth-order approximation up to second-order correction:
4.13
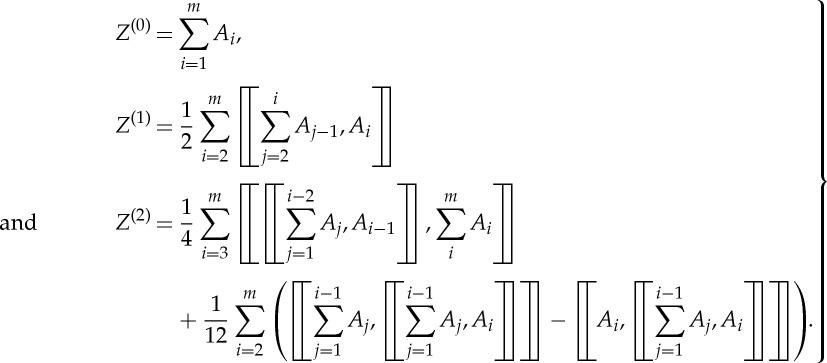
These formulae can be checked by taking *m*=3 and then compared with equations (4.8)–(4.10). Apart from an iteration of the BCH formula, another method to obtain the approximation for *Z* would be to expand each exponential function in the form of a Taylor series and collect the terms with the same order.

## 5. Corrector for high-order homogenization asymptotics

In this section, we introduce the corrector for the asymptotic error in the HOH algorithm, where a structure with thickness constant with respect to the frequency or the wavelength should be considered. Considering the multi-layered stack and its effective medium shown in figure 1, their transfer matrices should satisfy:
5.1
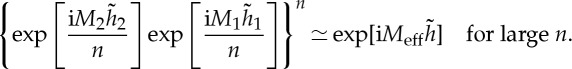
The BCH formula is still central to solve this problem, hence we recall its statement:
5.2
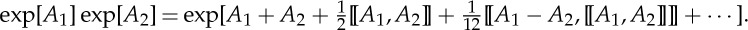
Let us take the first-order estimate in (5.2). This leads to the following error bound:

**Proposition 5.1**

*If A*_1_ *and A*_2_ *in* (*5.2*) *are bounded by the number* ∥*A*∥/2, *then*
5.3
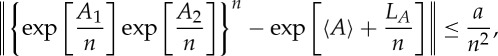
*with*
5.4
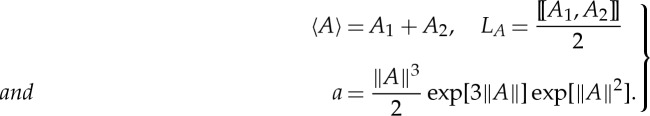

**Proof.**

Let *S*_*n*_ and *T*_*n*_ be defined by
5.5
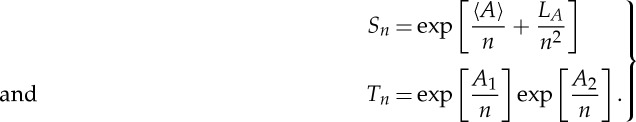
One can write
5.6
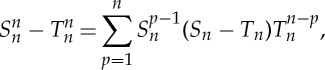
so that
5.7
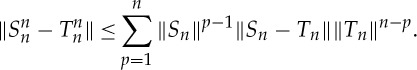
Straightforward upper bounds for *S*_*n*_ and *T*_*n*_ are:
5.8
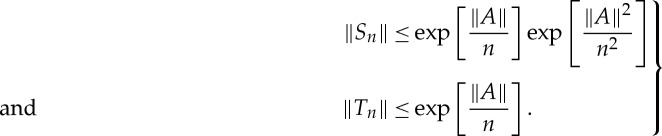
Then developing the exponential functions in (5.5) as a series,
5.9
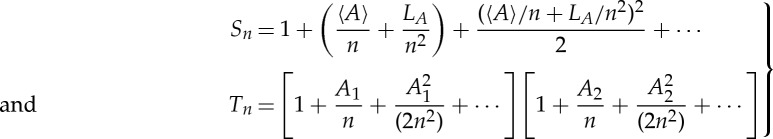
one has
5.10
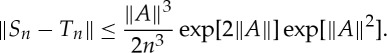
Substituting (5.8) and (5.10) into (5.7) provides us with the results (5.3)–(5.4). □

In periodic classical homogenization [7,34], only the leading order term is kept in the asymptotic procedure, hence the corrector is of order 1/*n*. Here, we find that:
5.11
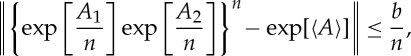
with
5.12
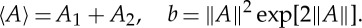
Let us emphasize that our iterative procedure amounts to adding more and more terms in the asymptotic expansion of classical homogenization and thus it improves the corrector of classical homogenization. Similar ideas have been implemented in the high-frequency homogenization recently developed by Craster *et al*. [11], however with no correctors being derived therein. It would also be interesting to see how randomness would affect our correctors: at order zero, the corrector is known to vary between *n*^−1/2^ and *n*^−1^ [35].

Furthermore, using the same proof process, we can also obtain the second-order estimate for the ansatz (5.2).

**Proposition 5.2**

*If A*_1_ *and A*_2_ *in* (*5.2*) *are bounded by the number* ∥*A*∥/2, *then*
5.13
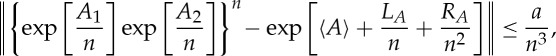
*with*
5.14
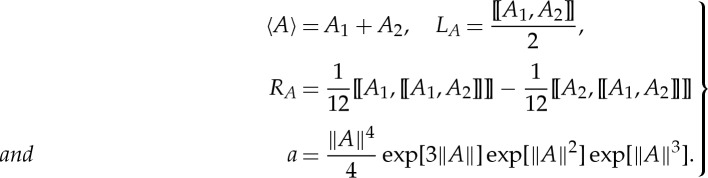

**Proof.**

Let *S*_*n*_ and *T*_*n*_ be defined by
5.15
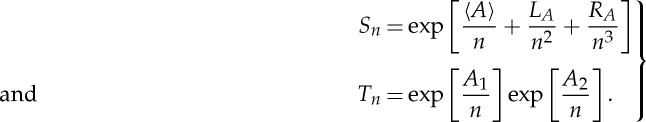

Upper bounds for *S*_*n*_ and *T*_*n*_ are once again straightforward:
5.16
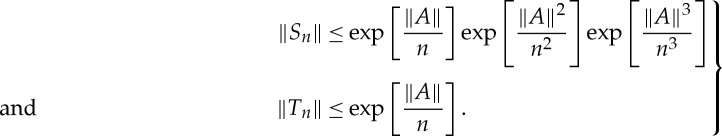

Moreover, developing the exponential function in (5.15) as a series,
5.17
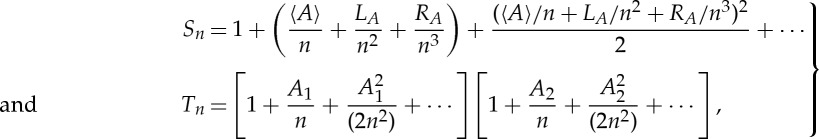
one has
5.18
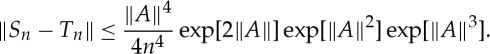
Substituting (5.16) and (5.18) into (5.7) leads to (5.13)–(5.14). □

It is noted that as the approximation order increases, the speed of the convergence defined by the difference between the transfer matrices of the multi-layers and the effective medium increases by a factor 1/*n*, hence it seems natural to conjecture that for the higher order approximation, the estimate between the transfer matrices of the multi-layers and the effective medium will be much more accurate with an error of 1/*n*^*p*^, with *p* the order taken in HOH approximation process.

Similarly, the asymptotic corrector can be derived for the stack with *m* layers. Here, we explore the corrector for HOH approximation in a multi-layered stack with three layers, where the permittivities are *ε*_1_, *ε*_2_, *ε*_3_ and thicknesses are 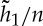, 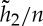, 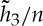 in a unit cell. Transfer matrices of the multi-layered stack approach the effective medium as follows:
5.19
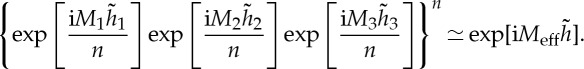
According to (4.1) and (4.8)–(4.10), the BCH formula leads to
5.20
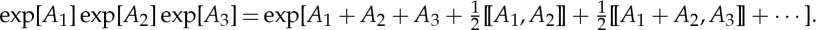
Taking the zeroth-order approximation as an illustration, we can state the following:

**Proposition 5.3**

*If A*_1_, *A*_2_ *and A*_3_ *in* (*5.20*) *are bounded by the number* ∥*A*∥/3, *then*
5.21
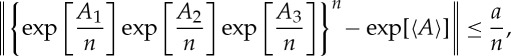
*with*
5.22
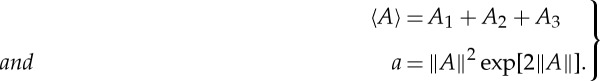

**Proof.**

Let *S*_*n*_ and *T*_*n*_ be defined by
5.23
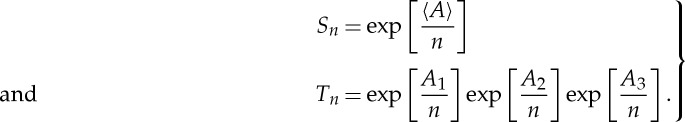
Equations (5.6) and (5.7) remain valid and upper bounds for *S*_*n*_ and *T*_*n*_ are
5.24
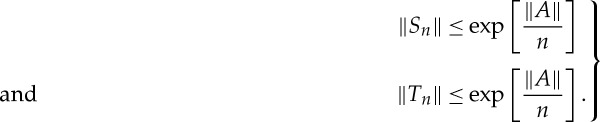
Then developing the exponential function as a series,
5.25
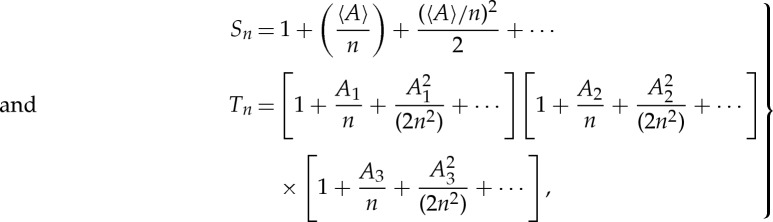
one has
5.26
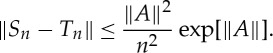
Substituting (5.24) and (5.26) into (5.7) leads to (5.21) and (5.22). □

The proof indicates that the corrector is of order *n*^−1^ when taking the classical homogenization (zeroth-order approximation) for a multi-layered stack with an alternation of three layers, this can be adopted for the *m* layers case. Correctors for higher order approximations as well as for a multi-layered stack consisting of an alternation of *m* layers can be obtained by the same algorithm.

## 6. Numerical calculations: dispersion law and transmission property

In this section, we numerically investigate the asymptotic estimate between the multi-layered stack and its effective medium obtained from HOH algorithm. We consider both their spectral (dispersion law) and scattering (transmission curve) properties. According to the previous analysis, the transfer matrix defined by the exponential function of matrix *M* is analytical, and it can be expanded as a Taylor series, taking the transfer matrix of the effective medium as an example,
6.1
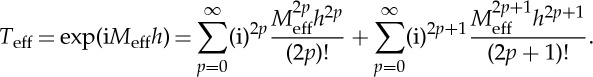
Considering an s-polarized incident wave, the column vector in (2.7) is defined by
6.2
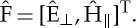
The matrix *M*_eff_ in (2.15) is a 2×2 matrix, and
6.3
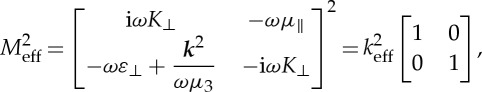
where
6.4
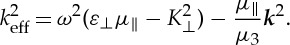
Plugging (6.3) into (6.1) and considering the Taylor series of the 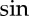
–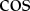 functions
6.5
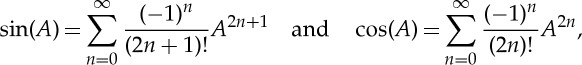
we obtain
6.6
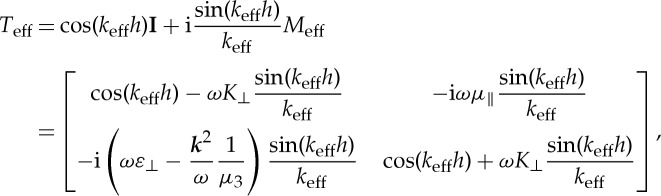
where **I** is the 2×2 identity matrix.

Similarly, the transfer matrix of the dielectric layer 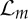 is
6.7
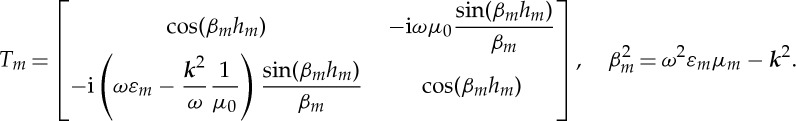
The transfer matrix *T* of the unit cell consisting of two dielectric layers is derived from the above expression as follows:
6.8
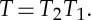


### (a) Dispersion law

A general expression for the dispersion law in a periodic structure is defined by the trace of the transfer matrix *T* of a single period [36–38]. As the eigenvalues and eigenvectors of *T* (and thus any power of *T*) are the Bloch phase factors and Bloch states of the periodic structure (in the limit of infinite *n*), respectively, further physical insight can be achieved in the single period matrix *T*. For a multi-layered stack with two layers, we have [39]
6.9
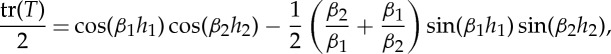
where *β*_*m*_ is defined in (??eq6.7), while for the effective medium,
6.10
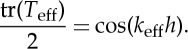
Here, *k*_eff_ defined by (6.4) can be obtained by substituting the expressions of the effective permittivity, permeability and magnetoelectric coupling, which are derived from the HOH algorithm.

**Figure 2.**
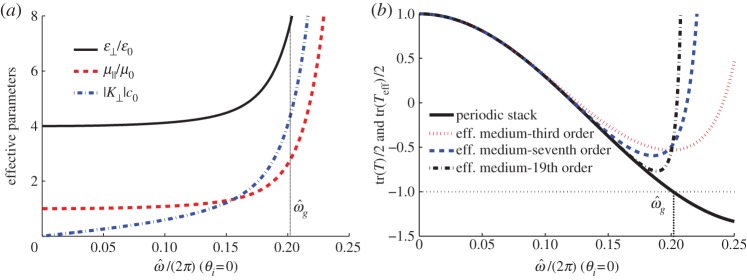
(*a*) Curves of the effective permittivity (solid line), permeability (dashed line) and magnetoelectric coupling (dotted-dashed line) versus normalized frequency at the 19th-order approximation and (*b*) dispersion laws of the multi-layered stack (solid line) and its effective medium at the third-order (dotted line), seventh-order (dashed line) and 19th-order (dotted-dashed line) approximations. HOH breaks down at 
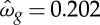
, lower edge of the first stop band. (Online version in colour.)

Considering a normal incident plane wave (***k***=**0**), the s-polarization and p-polarization coincide, as *ε*_∥_=*ε*_⊥_, *μ*_∥_=*μ*_⊥_, *K*_∥_=*K*_⊥_. Hence, we take an s-polarized incident wave as an example, and assume the two dielectric layers of the stack are Glass and Silicon, respectively; the relative permittivities are *ε*_1_/*ε*_0_=2 and *ε*_2_/*ε*_0_=12 and the filling fractions are *f*_1_=0.8 and *f*_2_=0.2. For the sake of illustration, in the HOH algorithm, we take the third-, seventh- and 19*th*-order approximations for the effective medium. The curves of the effective permittivity, permeability and magnetoelectric coupling at the 19*th*-order approximation versus normalized frequency are depicted in figure 2*a*, where 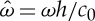, the expressions of these effective parameters are omitted here to save space. It is observed that all three curves increase with the frequency, wherein the effective permeability (dashed line) has values greater than 1, and effective magnetoelectric coupling (dotted-dashed line) is non-vanishing. In other words, artificial magnetism and magnetoelectric coupling can indeed be achieved from dielectrics through HOH asymptotics, as has been predicted in the theoretical analysis of §3.

Figure 2*b* shows the dispersion law of the stack as well as that of the effective medium at different orders approximation, which is obtained by substituting the expressions of the effective parameters into *k*_eff_ of (6.4) and then (6.10). The lower edge of the first stop band is denoted by 
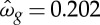
, where tr(*T*)/2=−1 [40]. It should be noted that a good agreement between the dispersion laws of the stack and the effective medium can be observed in the lower frequency band, and the asymptotics of these two curves can be improved by taking higher order approximation, e.g. the 19*th*-order approximation generates a curve (dotted-dashed line), which nearly coincides with that of the stack (solid line) from zero frequency (quasi-static limit) up to a normalized frequency around 0.18.

**Figure 3.**
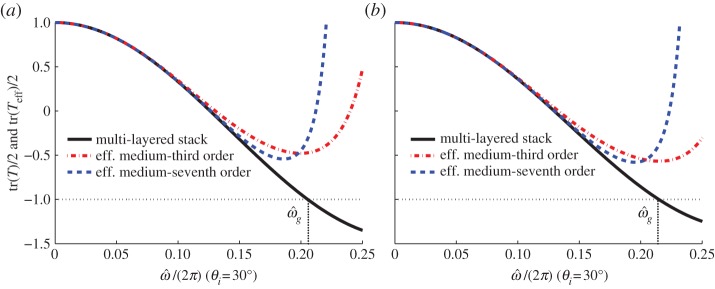
Dispersion laws of the stack (solid line) and its effective medium at the third-order (dotted-dashed line) and seventh-order (dashed line) approximations, under an oblique incident wave with *θ*_*i*_=30^°^, (*a*) s-polarization with band-gap edge 
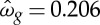
, (*b*) p-polarization with band-gap edge 
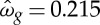
. (Online version in colour.)

Moreover, obliquely incident plane wave in s-polarization as well as in p-polarization are also analysed, wherein the same parameters of the stack are taken and the incident angle is *θ*_*i*_=30^°^, the dispersion laws of the multi-layered stack and the effective medium are shown in figure 3. The third- and seventh-order approximations are considered in the HOH algorithm, similarly, asymptotics improve in conjunction with higher order approximation for both s- and p-polarized waves.

### (b) Transmission property

**Figure 4.**
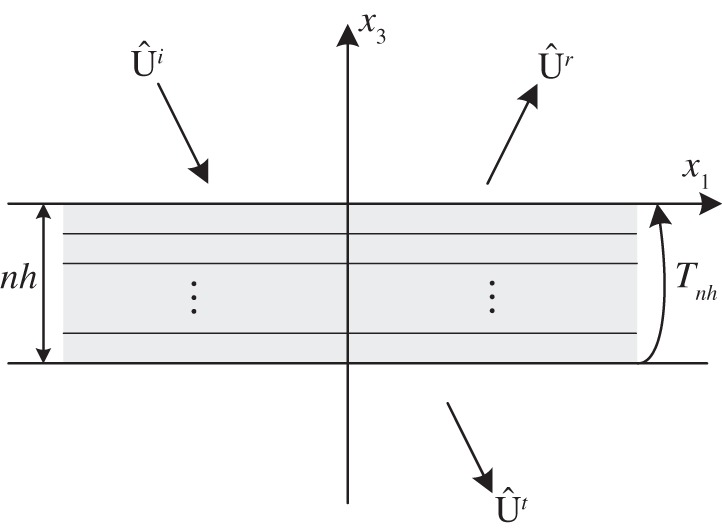
Schematic of the reflection and transmission for an incident wave on a stack of *n* identical unit cells, the thickness is *nh*, the upper and lower regions being vacuum.

Apart from the dispersion law, asymptotics between the transmission curves of the multi-layered stack and the effective medium is another important feature to be checked. Figure 4 shows a schematic of the reflection and transmission for an incident wave U^*i*^ on a stack of *n* identical unit cells, the thickness of which is denoted by *nh*; the upper and lower spaces of the stack are supposed to be vacuum with permittivity *ε*_0_ and permeability *μ*_0_.

**Figure 5.**
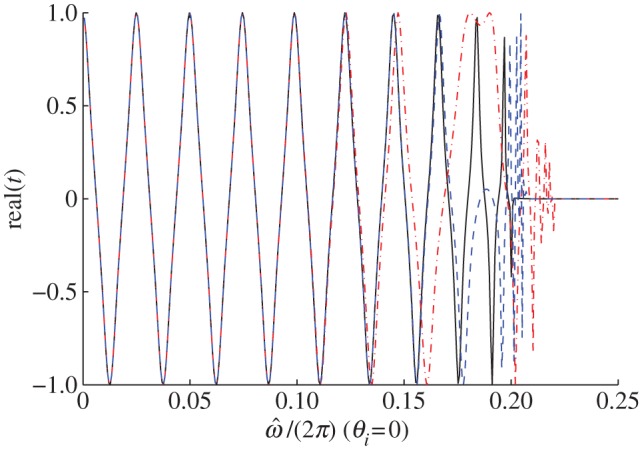
Transmission curves (*real*(*t*) versus normalized frequency) of the multi-layered stack (solid line) and its effective medium at the seventh-order (dotted-dashed line) and 19th-order (dashed line) approximation. (Online version in colour.)

We assume the incident wave in form of Fourier decomposition of (2.3) is
6.11
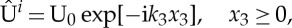
where 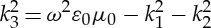, and *U*_0_ is the amplitude of the incident electromagnetic field. The reflected and transmitted waves are
6.12
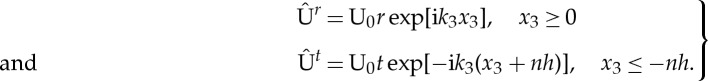
For a polarized incident wave, the column vectors 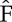 defined in (2.7) are
6.13
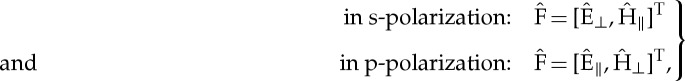
while for the regions (vacuum) above and below the stack (6.13) is simplified as
6.14
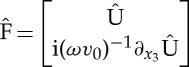
with
6.15
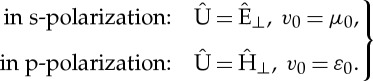
Assume that *T*_*nh*_ is the transfer matrix of the stack with thickness *nh*, we have
6.16
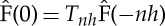
with
6.17
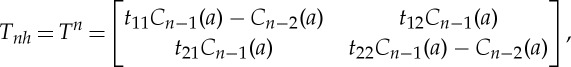
where *a*=(*t*_11_+*t*_22_)/2=tr(*T*)/2, *T* is the transfer matrix of the unit cell, and *C*_*n*_ are the Chebyshev polynomials of the second kind [41]
6.18
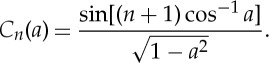
Again, if we consider an s-polarized plane wave in normal incidence, then 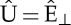 and 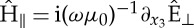, (6.16) leads to
6.19
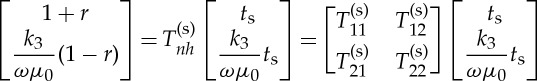
and the transmission coefficient is
6.20
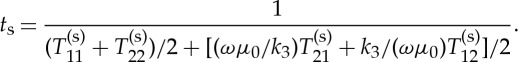
According to the duality between s- and p-polarization, one need only replace *μ*_0_ by *ε*_0_, as well as 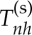 by 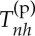 in (6.20) to obtain the transmission coefficient for p-polarization, e.g.
6.21
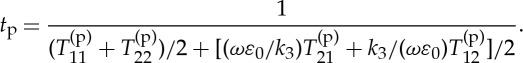
Let us consider again a multi-layered stack with *ε*_1_/*ε*_0_=2, *ε*_2_/*ε*_0_=12, *f*_1_=0.8 and *f*_2_=0.2 as an example, the thickness of the structure is supposed to be *nh* with *n* a constant, i.e. *n*=20, to run a numerical simulation in Matlab. Note that the s- and p-polarized incident waves coincide under a normal incidence, i.e. *t*_s_=*t*_p_=*t*. Applying *T*=*T*_2_*T*_1_ with *T*_*m*_ in (6.7) to (6.17) and (6.20), the transmission curve of the stack (solid line) is drawn in figure 5; while applying *T*=*T*_eff_ with *T*_eff_ in (6.6) to (6.17) and (6.20), the transmission curves of the effective medium at the seventh- (dotted-dashed line) and 19*th* (dashed line)-order approximations are obtained as shown in the same figure. The longitudinal coordinate of the figure is the real part of the transmission coefficient, while the abscissa is the normalized frequency.

It can be observed that the lower order approximation (dotted-dashed line) fits well with the curve of the stack (solid line) merely in the low frequency band; while an improved estimate (dashed line) can be achieved with higher order approximation (e.g. 19*th* order). In agreement with the dispersion law, the asymptotics between the two transmission curves of the stack (solid line) and the effective medium at the 19*th*-order approximation (dashed line) become invalid near the lower edge of the first stop band.

**Figure 6.**
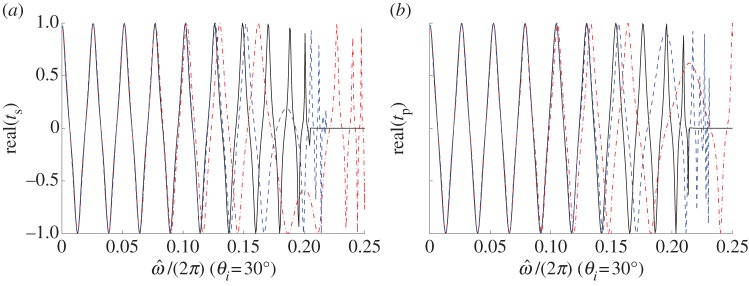
Transmission curves (*real*(*t*) versus normalized frequency) of the multi-layered stack (solid line) and its effective medium at the third-order (dotted-dashed line) and seventh-order (dashed line) approximations: (*a*) s-polarization and (*b*) p-polarization. (Online version in colour.)

Similarly, the transmission curves of the stack and the effective medium under an oblique incidence with *θ*_*i*_=30^°^ in s-polarization as well as p-polarization are shown in figure 6. Once more, the asymptotic approximation between the two transmission curves of the stack and the effective medium is quite good throughout the low frequency band, and improves with higher order approximation as it transpires from the dispersion laws of figure 3.

### (c) On logarithm of transfer matrix and analyticity

**Figure 7.**
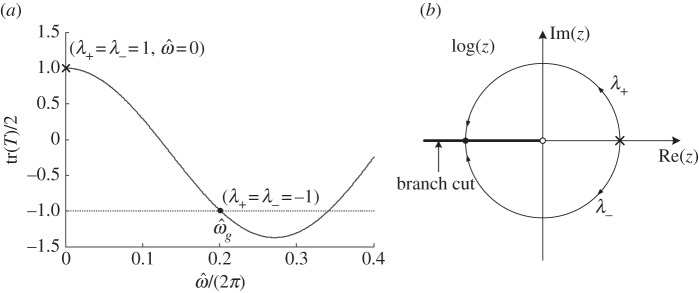
(*a*) Curve of tr(*T*)/2 versus normalized frequency for the multi-layered stack consisting of an alternation of two dielectric layers; the values of tr(*T*)/2 vary from +1 (cross sign) to −1 (filled point) in the lower pass band; while tr(*T*)/2=−1 defines the edge of the first stop band and (*b*) schematic of a branch of 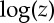, a region *G* (connected open subset in the complex plane) can be typically obtained by removing from 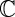 the negative real line 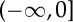. For the logarithm of *λ*_±_, we choose the upper and lower half circle paths for *λ*_±_ to approach −1 at the first edge of the first stop band (filled point) lying on the negative real axis.

From figures 3 to 6, it is noted that the asymptotics break down around the lower edge of the first stop band 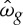 between the dispersion laws and the transmission curves of the multi-layered stack and its effective medium, no matter how high the order of approximation is. This invalidity is owing to the power-series expansion of *X*=i*M*_eff_*h* in (3.3) which diverges at 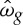. Indeed, we choose BCH formula to obtain the approximation for matrix *M*_eff_ and extract all the information required for effective permittivity, permeability and magnetoelectric coupling, by taking 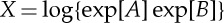 in (3.3). In complex analysis, a branch of 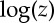 is a continuous function *L*(*z*) defined on a connected open subset *G* in the complex plane, such that *L*(*z*) is a logarithm of *z* for each *z* in *G* [42]. An open subset *G* is chosen as the set 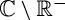 obtained by removing the branch cut (thick solid line) along the negative real axis and the branch point (empty point) *z*=0 from the complex plane, as shown in figure 7*b*.

On the other hand, the transfer matrix 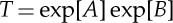 can be factorized by eigendecomposition as
6.22
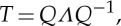
where *Q* is a square matrix consisting of the eigenvectors of *T*, and *Λ* is a diagonal matrix whose components are the eigenvalues (denoted by *λ*_±_) and
6.23
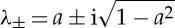
with *a*=tr(*T*)/2 the half trace of transfer matrix *T*. Furthermore, in order to derive the effective matrix *M*_eff_ for those effective parameters, we take the logarithm of *T*
6.24
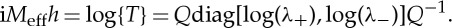
The curve of tr(*T*)/2 versus frequency is depicted in figure 7*a*, and one has *λ*_+_=*λ*_−_=*a*=+1 at the zero frequency (denoted by cross sign), while *λ*_+_=*λ*_−_=*a*=−1 at the edge of the first stop band 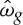 (denoted by filled point). In the lower frequency band, we choose *λ*_+_ and *λ*_−_ (starting from +1) that approach −1 via the upper half-circle path and the lower half-circle path, respectively, where the paths lie in the open subset *G*: 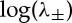 is always analytical and unique. However, at the edge of the stop band where *λ*_+_=*λ*_−_=*a*=−1 on the negative real axis, the logarithm is no longer analytical when its arguments meet at the branch cut of the logarithm: this implies that an expression of *M*_eff_ as a power series of the frequency *ω* has its radius of convergence bounded by 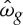. In other words, the effective parameters lose their efficiency for the approximation at frequencies higher than that of the first stop band, but they work just fine throughout the lower pass band. In order to achieve all frequency homogenization for a periodic structure, a new set of effective parameters (e.g. effective refractive index and surface impedance) should be introduced [33], where the analytical property of the transfer matrix in the complex plane is ensured.

## 7. Frequency power expansion of the transfer matrix

Although the function 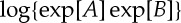 is no longer analytical at the lower edge of the first stop band, the transfer matrix 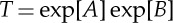 is analytical in the whole complex plane and can be approached by a power series at any frequency, a fact that will be numerically checked in this section. In mathematics, an exponential function can be approximated by a Taylor series as
7.1
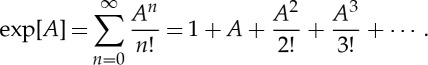
Hence, the transfer matrix *T* takes the form:
7.2
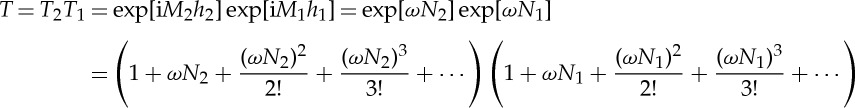
with notation *ωN*_*i*_=i*M*_*i*_*h*_*i*_. Let us expand and organize (7.2) in powers of *ω*,
7.3
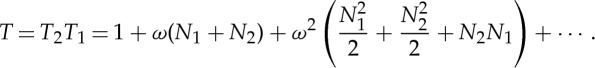
We collect the terms from *ω*^0^ to *ω*^*p*^ in (7.3) as an ansatz for *T*_eff_
7.4
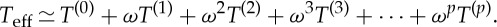
Obviously, with increasing *p*, the approximation in (7.4) becomes more accurate.

Considering a normal incident wave in s-polarization, the matrices *N*_*m*_ read as
7.5
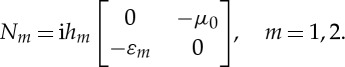
Substituting them into (7.4), the half trace of *T*_eff_ can be expressed as
7.6
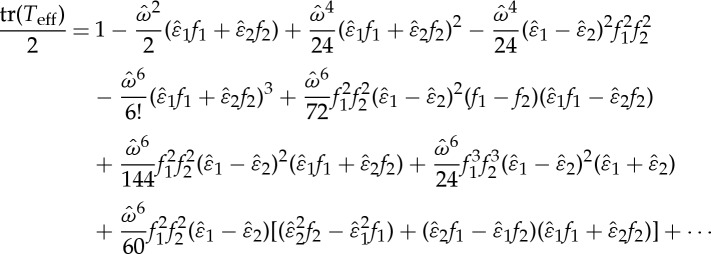
with the normalized frequency 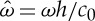 and 
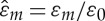
. It is noted that there are only terms containing even orders of 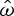, which is owing to the fact that the *T*^(2*p*+1)^ in (7.4) are all off-diagonal matrices with zero diagonal components.

**Figure 8.**
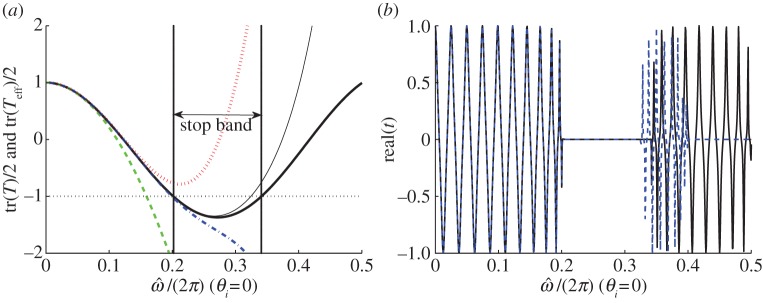
(*a*) Dispersion laws of the multi-layered stack (thick solid line) and the effective medium under a normal s-polarization incidence, the dashed, dotted, dotted-dashed and thin solid lines correspond to the approximation for *T*_eff_ in (7.4) by taking *p*=2,4,6,8. (*b*) Transmission curves (*real*(*t*) versus normalized frequency) of the stack (solid line) and the effective medium (dashed line) with *p*=8 in (7.4). Parameters of the dielectric layers of the stack are assumed to be the same as in §6. (Online version in colour.)

Dispersion laws are shown in figure 8*a*: the thick solid line represents the half trace of the transfer matrix for a multi-layered stack consisting of an alternation of two dielectric layers with the same parameters as assumed in §6, the dashed line, dotted line, dotted-dashed line and the thin solid line are tr(*T*_eff_)/2 with *p*=2,4,6,8 in (7.4) for *T*_eff_, respectively.

Upon inspection of the curve of the multi-layered stack (the thick solid line), it can be observed that the approximation for *T*_eff_ in (7.4) with *p*=2 (dashed line) is just efficient in a range of low frequency, while *p*=4 (dotted line) gives a sharper estimate for the multi-layered stack, but it totally misses the stop band. Moreover, if we push the approximation to *p*=6, the dispersion curves are nearly superimposed up to the edge of the first stop band, so well beyond the range of validity of classical homogenization [7]. However, the approximation with *p*=6 (dotted-dashed curve, which is always decreasing) breaks down at the lower edge of the stop band. In order to better approximate *T*_eff_, one needs to push the approximation to the next even power *p* (i.e. *p*=8, the thin solid line), which changes the curvature and gives a sharper estimate in the stop band region, although its intersection with the horizontal axis defines an approximate position for the upper edge of the stop band. This can be improved by taking a larger *p* in (7.4). Altogether, the larger the *p* in (7.4), the more accurate the approximation between the dispersion curves of the effective medium and that of the multi-layered stack.

Moreover, according to the expression of the transmission coefficient in (6.20), we take *p*=8 in (7.4) for *T*_eff_, the transmission curves of the stack (solid line) and the effective medium (dashed line) are compared in figure 8*b*. A good agreement between these two curves can be observed up to the first stop band, as predicted in figure 8*a*. Similar calculation can be applied to an oblique incidence. This demonstrates that the transfer matrix of the effective medium can be approached as a frequency power series at any frequency.

## 8. Concluding remarks

We provide a rigorous HOH algorithm for one-dimensional moderate contrast photonic crystals, where the period of the structure approaches the wavelength of the applied field. From an expression of transfer matrices in terms of exponential functions, Sophus Lie and BCH formulae are applied to produce HOH asymptotics. The analytical expressions of the effective parameters are derived for a stack with two layers in §3, where the artificial magnetism and magnetoelectric coupling effect are achieved in a moderate contrast periodic structure. In §4, we explore the extension of HOH algorithm to a stack with an alternation of *m* dielectric layers, and derive the expressions of the effective parameters for a centre symmetric stack: the magnetoelectric coupling vanishes while the artificial magnetism can be achieved with non-resonant periodic structures. Furthermore, the corrector for the approximation of a finite stack by its effective medium has been discussed in §5: the error estimate is of order 1/*n*^*p*^ with *p* the order of the approximation. Finally, based on the expressions of the effective parameters, we numerically validate our approximation method by comparing both the dispersion law and the transmission property of the stack and its effective medium in §6. The good agreement between these curves demonstrates that the HOH approximation is efficient throughout the lower pass band, while at the edge of the first stop band the logarithm function is no longer analytical. Finally, we investigate the approximation for the transfer matrix instead of the matrix *M*_eff_ of the effective medium by frequency power expansions. The dispersion laws as well as the transmission curves of both the multi-layered stack and the effective medium are explored in §7, and the excellent numerical agreement confirms that the transfer matrix of the effective medium can be approached by a power series at any frequency.
